# Soft-Tissue Sarcomas in Adults in Ecuador in the Period 2010–2020

**DOI:** 10.1155/2022/1391537

**Published:** 2022-12-26

**Authors:** Xavier Sánchez, Andrés Moreno Roca, Ruth Jimbo-Sotomayor, Luciana Armijos Acurio, Alfredo Viloria Cestari

**Affiliations:** ^1^Centro de Investigación Para la Salud en América Latina (CISeAL), Quito, Ecuador; ^2^Facultad de Medicina, Pontificia Universidad Católica del Ecuador, Quito, Ecuador; ^3^Servicio de Cirugía Oncológica, Hospital Carlos Andrade Marín, Quito, Ecuador

## Abstract

**Background:**

Soft-tissue sarcomas (STSs) are rare tumors; they represent 1% of all tumors in adults. There are new diagnostic techniques to differentiate tumor types, and surgery continues to be the most important treatment for STS.

**Methods:**

This cross-sectional study analyzed the morbidity and mortality caused by STS in adults between 2010 and 2020 using national databases.

**Results:**

A total of 8,393 patients hospitalized due to STS were reported. The total number of deaths in Ecuador due to STS was 7,088 over the last decade, the provinces of Pichincha and Guayas registered the highest number of cases, and the mortality rate was 1.2 to 2.0 per 100,000 people. It is noteworthy that the lowest point of registered cases was in 2012.

**Conclusion:**

Soft-tissue sarcomas are rare tumors in Ecuador. The morbidity and mortality caused by these tumors have not changed in the last decade. National studies are needed to determine the prevalence of this illness and study intervention to lower mortality.

## 1. Background

Soft-tissue sarcomas (STSs) are rare and heterogeneous tumors derived from the mesenchyme and represent about 1% of all malignancies in adults [1–3]. There are several subtypes of STS, and they can arise from cartilage, muscle, blood vessels, nerves, and fat [4].

STSs occur in 2–4 people per 100,000. The annual incidence of STS is approximately between 1.8 and 5.0 cases per 100,000 people per year [5]. The most common locations of these tumors are the lower limbs [6]. Genetic studies improve the diagnostic accuracy of STSs since they are often difficult to diagnose through morphological studies [7].

Surgery remains the primary treatment modality; however, chemotherapy and radiotherapy can be adjuvant treatments in certain STS [6]. Determining the clinical stage is essential to establish the best treatment option. Small, localized, and low-grade tumors have a good prognosis when treated with surgery. Large, wide, extended, and high-grade tumors that may not be suitable for surgery can be treated with adjunctive chemotherapy and radiotherapy [8]. Recurrences are common, and they are associated with the tumor size, histological grade and localization of the tumors [9].

The epidemiology of STS in adults is established by ICD-10 (International Statistical Classification of Diseases and Related Health Problems, 10th Revision) codes that are used to identify cases. This study aims to describe the current situation of patients who were hospitalized and the mortality due to STS in the period of 2010–2020 in Ecuador.

## 2. Methods

### 2.1. Study Setting and Design

This cross-sectional study analyzed the morbidity and mortality caused by STS in adults between 2010 and 2020 in Ecuador using national databases.

### 2.2. Data Sources

We analyzed the data available from the National Registry of Hospital Discharges and Deaths. This registry is of mandatory use in the National Health System of Ecuador, and the National Institute of Statistics and Census (INEC) is responsible for the validation of the information. These databases provide information from public and private health establishments.

### 2.3. Study Population

This study included adult patients over 18 years of age registered with ICD-10 codes related to STS (2020 WHO Classification of Tumors of Soft Tissue) between 2010 and 2020. The ICD-10 codes considered for the analysis were C47, C48, C49, C85.0, and C92.3 (Table 1). The variables included in the descriptive analysis were age, sex, place of residence, type of health establishment, length of hospital stay, and year of diagnosis.

### 2.4. Statistical Analysis

We performed a descriptive analysis of qualitative and quantitative variables related to morbidity and mortality. Results are presented through frequency distributions, proportions, rates, and measures of central tendency and dispersion. The statistical software used for the analysis was Stata Version 14.

## 3. Results

### 3.1. Morbidity

8393 cases of STS were reported to have been hospitalized during the period of the study.

General characteristics of the sample are displayed in Table 2. Distribution by gender was almost even between groups, 51% males versus 49% females. The most frequent diagnosis code reported for hospitalization was due to “malignant neoplasm of other connective and soft tissues” (C49) in 69.75% of the cases, followed by cases of malignant neoplasms of the retroperitoneum and peritoneum (C48) in 23.72% of the cases (Table 2).

Of all STSs, malignant neoplasms of peripheral nerves and autonomic nervous system (C47), lymphosarcoma (C85.0), and myeloid sarcoma (C92.3) represent only 4.43%, 1.29%, and 0.81% of cases, respectively.

The average length of hospital stay for all neoplasms was 6.2 days, ranging from 1 to 362 days.

**Figure 1:**
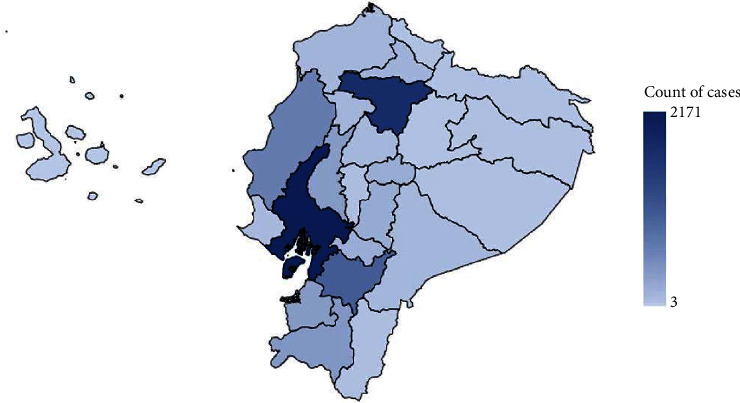
Number of cases of STS (STS: soft-tissue sarcoma).

The reported cases are evenly distributed among regions if we consider the population density. The provinces with more reported cases of STS were Guayas and Pichincha (Figure 1).

**Figure 2:**
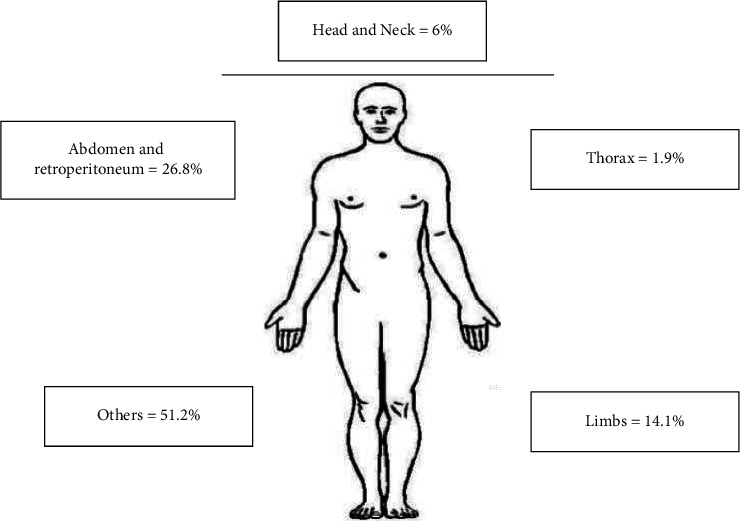
Anatomical location of neoplasms.

As shown in Table 3, the most common type of neoplasm registered was unspecified malignant neoplasm of connective and soft tissue (41.6%), followed by malignant neoplasms of the retroperitoneum and malignant neoplasms of connective and soft tissue of lower limbs, including hip (10%). Information of neoplasms by anatomical localization is depicted in Table 3, and Figure 2 shows a scheme of the anatomical location of the tumors reported in this study.

**Figure 3:**
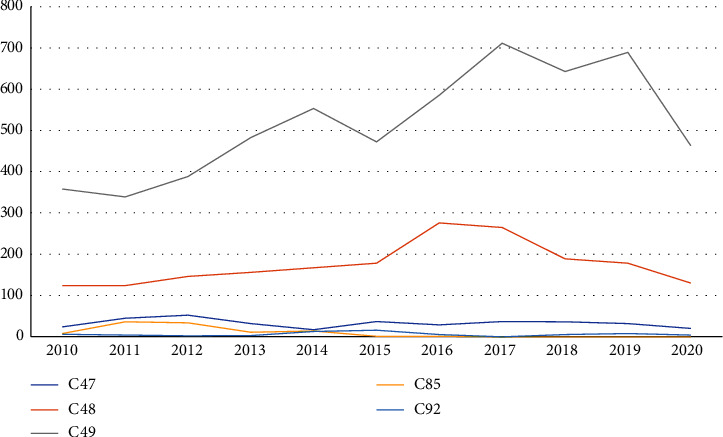
Evolution of neoplasms from 2010 to 2020 by ICD-10 codes (ICD-10: International Classification of Diseases, 10th Revision; C47: malignant neoplasm of peripheral nerves of the head, face, and neck; C48: malignant neoplasm of the retroperitoneum; C49: malignant neoplasm of other connective and soft tissues; C85: other specified and unspecified types of non-Hodgkin's lymphoma; C92: myeloid leukemia).

Between 2010 and 2020, there was a slight increase in tumors coded as C49. The rest of the neoplasms showed changes over the years but without a relevant increase over time (Figure 3).

### 3.2. Mortality

The total number of deaths in Ecuador due to soft-tissue sarcomas was 2904 over the last decade, in which 50.48% of deaths were in females and 49.52% of deaths were in males.

### 3.3. Number of Deaths per Year

**Figure 4:**
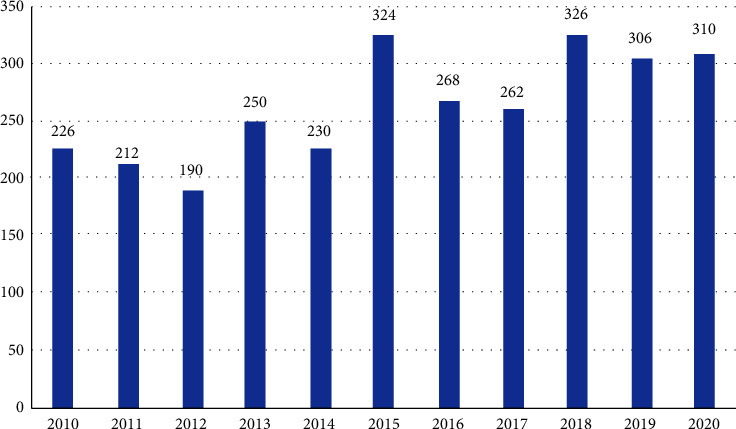
Total number of deaths per year due to STS.

The total number of deaths per year due to STS was relatively constant during the period of the study with an average of 264 cases per year, with the lowest number of cases reported in 2012 and the highest in 2018 (Table 4 and Figure 4).

The mortality rate per 100,000 people ranged from 1.2 to 2.0, with the lowest occurring in 2012 (Table 5).

## 4. Discussion

This is a large study of soft-tissue sarcomas in adults in Ecuador, with data from the National Registry of Hospital Discharges and Deaths in a 10-year cohort. The published data on the epidemiology of soft-tissue sarcomas are scarce; they represent less than 1% of malignant tumors, and little is known about their epidemiology or incidence patterns [10].

The data reported in several studies do not show a gender distribution; however, our results show an almost uniform distribution between genders, with a slight majority of patients being male (50.61%) and living in the highlands, results that are similar to those published by Bozzo et al. in Canada [11] in a five-year cohort study. However, these data are in contrast with those reported in Brazil in a sixteen-year cohort study where soft-tissue sarcomas were reported to be slightly higher in females [12]. The results of this study in this sense resemble those of a ten-year retrospective cohort study in Germany that reported an even distribution between genders [13]. We found that in the highland and coastal regions, there were more cases of soft-tissue sarcomas registered. This can be explained by the fact that the hospitals specializing in these pathologies are in these regions, where principal cities are located, and the population is more dense.

In contrast to other studies, we used information according to ICD-10. Soft-tissue sarcomas are relatively rare, but they can occur in almost any anatomic site [10]; however, we found that malignant neoplasm of unspecified connective and soft tissue sites was the most reported, in about 41% cases; this may be due to the manner of coding by the specialist. Nevertheless, although the literature shows that retroperitoneal soft-tissue sarcoma (STS) is relatively uncommon [14], we found that it was the second most reported diagnosis, accounting for 15% of cases. This contrasts markedly with the data reported by Saltus et al. [13], where the retroperitoneal location of these tumors was reported in only 6%, consistent with data reported by Toro et al. in the United States, including 26,758 cases between 1978 and 2001 [15]. Both studies reported that the most frequent anatomical locations of these tumors are the lower extremities, around 20%, which is in contrast with our findings in which this location was lower than retroperitoneal one, with 13.5% and 23.6%, respectively, if we consider all ICD-10 codes.

A local study [16] that included 71 patients with a diagnosis of soft-tissue sarcomas in a single site, specialized hospital in Ecuador, reported that 67.6% of tumors were in the extremities. This finding is distinct from our study in which only 13.6% were in the extremities; nevertheless, the report of many cases as “unspecified” site could correspond to the location in the extremities.

During the 2010–2020 period, there was a slight increase in diagnoses of STS coded with ICD-C49 (connective, subcutaneous, and other soft tissues). It is comparable with the study by Alorjani et al. published in 2021 [17], which showed that 58% of the cases were ICD-C49. Yang et al. [18] also found that the most frequent diagnosis was ICD-C49 in 22.61% cases.

We encountered a mortality rate of 1.2 to 2.0 per 100,000 habitants due to STS in Ecuador. A similar mortality rate was reported by Saltus et al. in 2018 [13], with an average annual mortality rate of 2.31 per 100,000 habitants. Our report also is comparable with the data from the United States National Cancer Institute with a mortality rate of 1.3 per 100,000 per year in 2021 [19].

The most important limitation to this study is that there is no adequate definition of ICD-10 coding for patients with soft-tissue sarcomas, since some patients are coded with unspecific diagnoses. In addition, the variables considered in our study come from national data sources that do not include sociodemographic and clinical variables necessary for a better understanding of this pathology.

## 5. Conclusion

This study has analyzed the most recent data on hospital discharges and mortality from soft-tissue sarcomas in the Ecuadorian population. Hospital discharges and mortality rates due to soft-tissue tumors have not seen major changes in Ecuador in the last decade.

## Figures and Tables

**Table 1:** ICD-10 codes related to STS.

ICD-10 code	Description
C47	Malignant neoplasms of peripheral nerves and autonomic nervous system
C48	Malignant neoplasms of the retroperitoneum and peritoneum
C49	Malignant neoplasm of other connective and soft tissues
C85.0	Lymphosarcoma
C92.3	Myeloid sarcoma

ICD-10: International Classification of Diseases, 10th Revision; STS: soft-tissue sarcoma.

**Table 2:** General characteristics of patients with STS.

Variable	ICD-10				
	C47	C48	C49	C85.0	C92.3
Days of hospitalization	5.73	7	5.9	5.19	6.64
Sex					
Male	239 (64.9)	940 (47.3)	2973 (50.7)	61 (58.1)	35 (50.7)
Female	129 (35.1)	1047 (52.7)	2891 (49.3)	44 (41.9)	34 (49.3)
Region					
Coast	133 (36.4)	935 (47.2)	259 (44.4)	57 (54.3)	44 (64.7)
Highlands	198 (54.2)	998 (50.4)	3015 (51.5)	46 (43.8)	23 (33.8)
Amazon	34 (9.3)	48 (2.4)	243 (4.1)	2 (1.9)	1 (1.5)
Insular	0 (0)	0 (0)	3 (0.1)	0 (0)	0 (0)

ICD-10: International Classification of Diseases, 10th Revision; STS: soft-tissue sarcoma; C47: malignant neoplasm of peripheral nerves of the head, face, and neck; C48: malignant neoplasm of the retroperitoneum; C49: malignant neoplasm of other connective and soft tissues; C85.0: other specified and unspecified types of non-Hodgkin's lymphoma; C92.3: myeloid sarcoma.

**Table 3:** Details of neoplasms by three-digit ICD-10 codes.

Type of neoplasm	Description	*N*	%
Head and neck		500	6.0
C470	Malignant neoplasm of peripheral nerves of the head, face, and neck	49	0.6
C472	Malignant neoplasm of peripheral nerves of lower limbs, including hip	59	0.7
C490	Malignant neoplasm of connective and soft tissue of the head, face, and neck	392	4.7
Thorax		157	1.9
C473	Malignant neoplasm of peripheral nerves of the thorax	16	0.2
C493	Malignant neoplasm of connective and soft tissue of the thorax	141	1.7
Abdomen and retroperitoneum		2249	26.8
C474	Malignant neoplasm of peripheral nerves of the abdomen	18	0.2
C480	Malignant neoplasm of the retroperitoneum	1281	15.3
C481	Malignant neoplasm of specified parts of the peritoneum	113	1.3
C482	Malignant neoplasm of the peritoneum, unspecified	422	5.0
C488	Malignant neoplasm of overlapping sites of the retroperitoneum and peritoneum	167	2.0
C494	Malignant neoplasm of connective and soft tissue of the abdomen	248	3.0
Limbs		1183	14.1
C471	Malignant neoplasm of peripheral nerves of upper limbs, including shoulder	52	0.6
C491	Malignant neoplasm of connective and soft tissue of upper limbs, including shoulder	297	3.5
C492	Malignant neoplasm of connective and soft tissue of lower limbs, including hip	834	10.0
Others		4289	51.2
C475	Malignant neoplasm of peripheral nerves of the pelvis	39	0.5
C476	Malignant neoplasm of peripheral nerves of the trunk, unspecified	17	0.2
C478	Malignant neoplasm of overlapping sites of peripheral nerves and autonomic nervous system	12	0.1
C479	Malignant neoplasm of peripheral nerves and autonomic nervous system, unspecified	105	1.3
C495	Malignant neoplasm of connective and soft tissue of the pelvis	263	3.1
C496	Malignant neoplasm of connective and soft tissue of the trunk, unspecified	103	1.2
C498	Malignant neoplasm of overlapping sites of connective and soft tissue	92	1.1
C499	Malignant neoplasm of connective and soft tissue, unspecified	3486	41.6
C850	Other specified and unspecified types of non-Hodgkin's lymphoma	103	1.2
C923	Myeloid sarcoma	69	0.8

ICD-10: International Classification of Diseases, 10th Revision.

**Table 4:** Mortality per year due to STS by gender.

	2010	2011	2012	2013	2014	2015	2016	2017	2018	2019	2020
C47	10	6	4	5	4	8	8	3	6	11	3
Male	4	3	3	2	2	6	4	2	3	6	3
Female	6	3	1	3	2	2	4	1	3	5	0
C48	39	27	28	56	45	59	38	48	60	49	56
Male	17	11	14	21	23	29	18	25	26	18	24
Female	22	16	14	35	22	30	20	23	34	31	32
C49	64	73	63	64	66	95	88	80	97	93	96
Male	38	43	36	37	31	41	47	51	45	38	48
Female	26	30	27	27	35	54	41	29	52	55	48
Total	226	212	190	250	230	324	268	262	326	306	310

STS: soft-tissue sarcoma.

**Table 5:** STS mortality rate per 100,000 people.

Year	Mortality rate per 100,000 people
2010	1.5
2011	1.4
2012	1.2
2013	1.6
2014	1.4
2015	2.0
2016	1.6
2017	1.6
2018	1.9
2019	1.8
2020	1.8

## Data Availability

Data for this study were obtained from the National Registry of Hospital Discharges and Deaths published as open access from INEC (National Institute of Statistics and Census). These data could be accessed from https://www.ecuadorencifras.gob.ec/estadisticas/.
